# Risk Factor Prediction Model for Catheter-Associated Bloodstream Infections (CABSIs) in Midline and Central Venous Catheters: A Cohort Follow-Up Study

**DOI:** 10.3390/jcm15093243

**Published:** 2026-04-24

**Authors:** Elisabeth Lafuente-Cabrero, Roser Terradas-Robledo, Anna Civit-Cuñado, Diana García-Sardelli, Carla Molina-Huerta, Ines Gerez-Acevedo, Dolors Giro-Formatger, Laia Lacueva-Perez, Cristina Esquinas, Avelina Tortosa

**Affiliations:** 1 Department of Infusion and Vascular Access Nurse, Nursing Care Research, Hospital del Mar Research Institute, Hospital del Mar, 08003 Barcelona, Spain; elafuente@hmar.cat (E.L.-C.); acivit@hmar.cat (A.C.-C.); dgarciasardelli@hmar.cat (D.G.-S.); cmolina@hmar.cat (C.M.-H.); 2 Department of Nursing Methodology, Quality and Research, Hospital del Mar, 08003 Barcelona, Spain; mterradas@hmar.cat (R.T.-R.); mgiro@hmar.cat (D.G.-F.); llacueva@hmar.cat (L.L.-P.); 3 Department of Infusion and Vascular Access Nurse, Hospital Doctor Josep Trueta, 17007 Girona, Spain; igereza.girona.ics@gencat.cat; 4 Department of Public Health, Mental Health and Maternal and Child Health Nursing, Faculty of Nursing, University of Barcelona (UB), 08007 Barcelona, Spain; 5 Department of Basic and Clinical Nursing, Faculty of Nursing, University of Barcelona (UB), 08907 Barcelona, Spain; atortosa@ub.edu

**Keywords:** peripherally inserted central catheter (PICC), centrally inserted central catheter (CICC), midline, catheter-associated bloodstream infection (CABSI), predictive model, risk factor, nurse, vascular access team (VAT), infection control

## Abstract

**Background**: Venous catheter placement is the most common invasive procedure performed in hospitals. Despite their widespread use and importance in healthcare, these devices can cause complications such as catheter-associated bloodstream infections (CABSIs). Although several studies have investigated potential risk factors, including sociodemographic, medical history, and clinical variables, the results remain inconsistent and inconclusive. **Objectives**: The aim of this study was to identify independent risk factors for CABSIs and to develop and validate a predictive model for CABSIs in patients with midline catheters, centrally inserted central catheters (CICCs), and peripherally inserted central catheters (PICCs). **Methods**: We conducted an observational cohort follow-up study including hospitalized patients with a CICC, PICC, or midline catheter between January 2016 and March 2022. Devices were randomly assigned to derivation (n = 6036) and validation (n = 1549) cohorts. Candidate predictors with *p* < 0.25 in univariate analysis entered a multivariable logistic regression model, and final variables were selected by backward stepwise regression. Performance in the validation cohort was assessed by calibration and discrimination using the Hosmer–Lemeshow test and AUC. **Results**: The prevalence of CABSIs in the derivation cohort was 1.8%. Independent risk factors for CABSIs included tracheostomy, a history of bacteremia within 3 months before catheter placement, the presence of a synchronous central catheter, active oncohematological disease, and having received total parenteral nutrition (TPN). The presence of these five variables increased the probability of CABSIs to 42.1%. The final model demonstrated good predictive performance with an area under the curve (AUC) of 0.73 in the derivation cohort and 0.77 in the validation cohort. Decision curve analysis showed that the predictive model offered a greater net clinical benefit than the “treat-all” or “treat-none” strategies among threshold probabilities between 0.5% and 5%. **Conclusions**: The model can help identify high-risk patients, guide risk-based clinical decisions, reduce unnecessary catheter use, and support infection prevention and antimicrobial stewardship.

## 1. Introduction

Vascular devices are an essential component of modern healthcare and represent the most commonly performed invasive procedures in hospitals. In Spain, a study reported that 76.57% of hospitalized patients had a peripheral venous device (PVD), while 12.34% had a central venous device (CVD) [1]. In the United States, more than 330 million PVDs and 5 million CVDs are inserted annually [2,3]. Despite their high prevalence, vascular devices can lead to complications, some of which can be severe or even fatal [4,5,6,7].

One of the most common complications, with a significant impact on the comorbidity of patients with vascular devices, is catheter-associated bloodstream infection (CABSI). The Spanish National Prevalence Study of Nosocomial Infections (EPINE) has reported that 16.23% of nosocomial infections are due to CABSI [1], with a mortality rate of 9.4% [6]. In the USA, approximately 30,100 CABSI episodes occur annually among critically ill patients [5], with attributable mortality ranging from 12% to 25% [7]. Given the severity of this issue and the known pathogenesis of CABSIs, numerous preventive strategies have been established to standardize the insertion, management, and care of vascular devices, as outlined by various national and international scientific societies [5,7,8]. In addition, the Association for Professionals in Infection Control and Epidemiology (APIC, 2015) has stated that most CLABSIs can be prevented through the implementation of evidence-based strategies currently available to healthcare professionals [4]. Identifying risk factors would allow tailored monitoring, care, and maintenance protocols, as well as the incorporation of technology and new devices to reduce individual patient risk. Numerous studies have investigated risk factors for CABSIs across different patient populations, including general hospitalized, ICU, surgical, and oncology patients [9,10,11]. However, findings have been heterogeneous and have not allowed consistent identification of independent predictors [9,12,13,14]. In addition, to our knowledge, no predictive tool has been developed to estimate CABSI risk before catheter insertion in patients with midline catheters, CICCs, and PICCs. Therefore, this study aimed to identify independent risk factors for CABSIs and to develop and validate a predictive model in this population.

## 2. Methods

### 2.1. Study Design, Setting and Patient Sample

This was a cohort follow-up study that included all hospitalized patients aged 18 years or older with a CICC, PICC or midline catheter between January 2016 and March 2022. The length of follow-up for each patient was defined from catheter insertion until removal. This study was performed in a university hospital in Barcelona, Spain.

This study was conducted and reported in accordance with the STROBE (Strengthening the Reporting of Observational Studies in Epidemiology) guidelines for cohort studies. All catheters implanted in this study were from BD (Becton, Dickinson and Company, Franklin Lakes, NJ, USA) or ARROW (Teleflex Incorporated, Morrisville, NC, USA).

### 2.2. Inclusion and Exclusion Criteria

The inclusion criteria were patients 18 years of age who had a CICC, PICC, or midline catheter between January 2016 and March 2022. The exclusion criteria included patients with a CICC, PICC, or midline catheter for less than 24 h and outpatients. Patients hospitalized in 2020 were also excluded due to the COVID-19 pandemic because the exceptional context of the COVID-19 pandemic may have affected the completeness and comparability of clinical records and the consistency of usual venous catheter care processes.

### 2.3. Catheter Insertion Procedure

All venous catheters were monitored until withdrawal. The insertion, maintenance, and withdrawal procedures for the venous catheters included in the study were performed according to the protocols of our institution, which are based on the guidelines of the Centers for Disease Control and Prevention/National Healthcare Safety Network (CDC/NHSN) and the Infusion Nursing Society (INS) [15]. PICCs and midline catheters were inserted by the Vascular Access Team (VAT) at our center, composed of qualified nurses with expertise in vascular access. The type of catheter inserted was chosen based on clinical recommendations and guidelines [15]. PICCs and midline catheters were inserted at the patient’s bedside. For CICC insertion, the procedure was performed in the ICU, medical anesthesia, or general surgery department in a critical care bay or operating room. All catheters were inserted under ultrasound guidance, and the position was confirmed by the professional performing the procedure. Nursing staff responsible for the patient managed the maintenance and care of the venous catheters, as well as their withdrawal, following institutional protocols.

### 2.4. Variables and Data Collection

Data were obtained from electronic clinical records through the IMAS (IMASIS) hospital information system. Variables were collected in a database designed for this purpose and accessible only to study investigators. Follow-up and registry of the variables were conducted by the VAT.

Collected variables included sociodemographic data, patient history, the Charlson comorbidity index adjusted for age [16], analytical data (complete blood count, biochemical parameters, and inflammation: C-reactive protein [CRP] and procalcitonin), unit of hospitalization at catheter placement, and whether the patient was admitted for specific care (hemodialysis, emergency surgical intervention, ICU/resuscitation (ICU/RES), invasive mechanical ventilation, or septic shock during catheter use). In addition, variables related to pharmacotherapy administered on the day of catheter placement, days of catheter dwell time, and the days from admission to catheter insertion were collected. The following catheter-related variables were also gathered: type of catheter implanted, characteristics of the catheter and its insertion, patient history related to catheters, analytical parameters on the day of catheter insertion, and the microorganism causing CABSI.

The same predefined variables, outcome definitions, and data collection criteria were applied consistently throughout the entire study period.

### 2.5. Definitions and Measurement Tools

The main study variable was the presence of CABSI after catheter insertion. The CABSI definition used in this study, as described by the INS in 2021 [15], included midline catheters, which are usually excluded from the definition of central line-associated bloodstream infection (CLABSI). CABSI was defined according to the criteria established by the CDC/NHSN [17].

All implanted PICCs were made of third-generation power-injection polyurethane. A PICC is defined as a venous catheter inserted peripherally with the distal point in the lower third of the superior vena cava (SVC) [15]. The CICCs were made of second-generation polyurethane impregnated with an antimicrobial. A CICC is defined as a catheter inserted in a central vein, with the distal point in the SVC [15]. A midline catheter made of second-generation polyurethane is a peripheral venous catheter with a length of 8–25 cm, with the distal point placed at the level of the axilla [15,18].

### 2.6. Ethical Considerations

Informed consent was waived due to the retrospective nature of data collection and the use of anonymized clinical data. Although the data were obtained from pre-existing clinical records, the study was designed as the follow-up of a cohort to assess the occurrence of CLABSIs over time. Data corresponding to the period from January 2016 to March 2022 were collected retrospectively from existing clinical records. The study was conducted in accordance with the ethical principles of the Declaration of Helsinki and was approved by the Clinical Research Ethics Committee of the Hospital del Mar Medical Research Institute (CEIC2018/8113/I), with approval granted on 21 September 2018.

### 2.7. Statistical Analysis

The unit of analysis was the catheter. To develop a predictive model for CABSIs, the total sample size was randomly divided into a derivation cohort comprising 80% of the patients (n = 6036) and a validation cohort comprising 20% of the patients (n = 1549). All analyses and the predictive model were performed using the derivation cohort, and the predictive model was subsequently internally validated in a randomly split validation cohort. The characteristics of the catheters and the patients in the study cohorts were compared.

Qualitative variables are expressed as absolute frequencies and percentages, while quantitative variables are expressed as the mean and standard deviation. The Kolmogorov–Smirnov test was used to evaluate the normality of the distributions.

Several indices, such as the neutrophil-to-lymphocyte ratio (NLCR), were calculated [17]. The variables of interest were compared based on the presence of CABSIs. For quantitative variables, the Student t-test was performed. The chi-square test (or Fisher’s exact test for frequencies < 5) was used to compare categorical variables.

Variables with a *p*-value < 0.25 in the univariate analysis were considered for inclusion in the multivariable model, following recommended strategies to avoid excluding potentially important predictors [19]. To reduce the risk of overfitting, the number of predictors included in the final model was restricted according to the number of events. The final selection of variables was made using backward regression (likelihood ratio) (Pin < 0.05; Pout > 0.10) [19]. The multiple imputation method was used for missing data. The data are expressed as odds ratios (ORs), 95% confidence intervals (CIs) and *p* values.

The β coefficients from the final multivariable model were used to derive the weighted score. Each coefficient was divided by the smallest coefficient in the model and rounded to the nearest integer to assign points proportional to the strength of association. In addition, a simplified non-weighted score was created by assigning one point to each predictor.

The Hosmer–Lemeshow goodness-of-fit test evaluated the overall fit of the model. Receiver operating characteristic (ROC) curves were developed to calculate the predictive power of the final model. The combination of predictors in the final model was used to calculate the probability of CABSIs. A final score was developed based on previously published and validated risk assessment tools [12]. Cut-off values were assigned to create a tool with a point system from 0 to 15, with individual points reflecting the relative magnitude of the regression coefficients in the model. The final model was validated using the validation cohort.

Missing data were handled using multiple imputation. The proportion of missing data was low (<5%) for most variables, although the number of lumens showed slightly higher levels of missingness (5.7% in the derivation cohort and 6.4% in the validation cohort), and serum albumin presented a higher proportion of missing data (66% in both cohorts). Five imputed datasets were generated using regression-based models appropriate for each variable type (logistic regression for categorical variables and linear regression for continuous variables). Plausible ranges were defined for continuous variables to ensure realistic imputations. As results were consistent across imputations, analyses were conducted on one representative imputed dataset. A *p* value < 0.05 was considered statistically significant for all tests. The R Studio (V4.3) statistical package was used for the analyses.

## 3. Results

### 3.1. Descriptive Statistics

A total of 7585 venous catheters were inserted: 6036 in the derivation cohort and 1549 in the validation cohort. There were no significant differences between the two cohorts except in history of cerebrovascular disease, catheter length, length of hospital stay prior to catheter insertion, and procalcitonin values; overall, both cohorts were comparable (Table 1 and Table 2). Of the catheters inserted, 1.8% developed CABSIs according to CDC/NHSN criteria.

The mean age of the patients in the derivation cohort was 71 (SD, 16) years, and 59% of the catheters were implanted in men. Catheter insertion took place in a conventional hospitalization unit in 58.9% of the patients. The most common antecedent was oncological disease in 31.4% of the patients, followed by diabetes mellitus in 29.5%. CABSIs had occurred within 3 months prior to the insertion of a new catheter in 15.2% of the patients.

The mean length of hospitalization until venous catheter insertion was 10.91 (SD, 28.89) days and the mean dwell time was 6.30 (SD, 9.24) days. Venous catheter insertions were most frequent in the fall (27.1% of all insertion).

The most frequently administered pharmacotherapy was TPN, used in 20% of catheters. The mean number of catheter manipulations within a 24 h period was 13 (SD, 13.89).

The most frequently used venous catheter was a CICC in 49.1% of insertions. In 5.6% of insertions, a synchronous central catheter was present, and 40.3% of patients had a history of another venous catheter (CICC, PICC or midline catheter) during the same hospital admission. The mean number of short peripheral lines placed prior to catheter insertion was 1.73 (SD, 2.56).

With respect to laboratory parameters at the time of insertion, mean values were as follows: albumin 3.08 (0.71) g/dL, eosinophils 0.13 (0.22) u/µL, leukocytes 12.11(7.9) u/µL, NLCR 11.67 (15.52), neutrophils 11.84 (15.55) u/µL, CRP 10.60 (11.77) mg/dL and procalcitonin 13.28 (45.59) ng/mL (Table S1).

### 3.2. Risk Factors for the Presence of CABSI (Derivation Cohort)

In the validation cohort, CABSI (N = 1549) was observed in 29 patients (1.8%). Bivariate analysis showed that patients who developed CABSI more frequently experienced bacteremia in the previous three months (33.6% vs. 14.9%, *p* < 0.001), and had a history of active oncohematological disease (10.3% vs. 4.7%, *p* = 0.008). Patients with CABSIs were also more frequently receiving hemodialysis (10.3% vs. 3.5%, *p* = 0.002) and invasive mechanical ventilation (24.3% vs. 11.9%, *p* > 0.001), and had a tracheostomy (19.6% vs. 10.3%, *p* = 0.002). In this group, both the length of hospital stay prior to catheter insertion (37.49 vs. 10.43, *p* < 0.001) and catheter dwell time (18.84 vs. 8.84 days, *p* = 0.004) were also longer. Among pharmacological therapies, peripheral parenteral nutrition (6.5% vs. 2.6%, *p* = 0.025), TPN (55.1% vs. 19.4%, *p* < 0.001), and parenteral serum therapy (27.1% vs. 19.4%, *p* = 0.048) were more frequent in the CABSI group.

Regarding catheter characteristics, patients with CABSIs had a greater number of lumens implanted (2.69 vs. 2.34, *p* = 0.002) and longer catheter lengths (30.49 vs. 27.17 cm, *p* = 0.005). They were also more likely to have a synchronous central catheter (18.7% vs. 5.3%, *p* < 0.001), a central catheter during the same hospital stay (59.8% vs. 40.0%, *p* < 0.001) and a higher number of peripheral lines implanted prior to the insertion of a CICC, PICC, or midline catheter (3.31 vs. 1.7, *p* < 0.001).

In regard to analytical parameters, only albumin levels were significantly higher in the NO CABSI group (3.08 vs. 2.7 g/dL, *p* = 0.001) (Table S2).

### 3.3. Prognostic Equation for CABSIs

**Figure 1:**
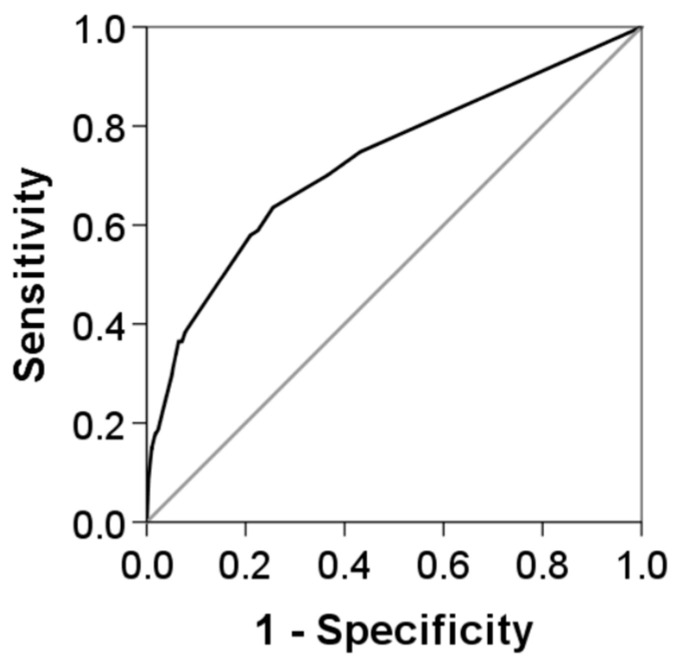
Predictive performance of the final multivariate model for the presence of CABSIs in the derivation cohort.

The final logistic regression model identified five factors independently associated with CABSIs: tracheostomy (OR 1.85, 95% CI 1.12–3.06, *p* = 0.017), bacteremia within 3 months prior to catheter insertion (OR 2.20, 95% CI 1.43–3.37, *p* < 0.001), the presence of a synchronous catheter (OR 2.25, 95% CI 1.32–3.85, *p* = 0.003), active oncohematological disease (OR 2.56, 95% CI 1.33–4.92, *p* = 0.005), and receipt of TPN (OR 4.47, 95% CI 3.00–6.64, *p* < 0.001). The weighted score derived from this model assigned the highest value to TPN (15 points). This final model obtained good goodness-of-fit with the Hosmer–Lemeshow test (*p* = 0.735) and a strong predictive ability for CABSIs (AUC = 0.73, 95% CI 0.67–0.78) (Table 3 and Table 4, and Figure 1). Based on the final model’s risk estimates, the (negative predictive value) NPV ranged from 99.2% to 98.8%, whereas the PPV ranged from 3% to 9%.

The prognostic equation estimated that the probability of CABSIs was 0.7% in patients with none of the identified risk factors. Among patients with only one risk factor, the probabilities were 1.3% for tracheostomy, 1.5% for bacteremia within 3 months prior to catheter insertion, 1.5% for a synchronous central catheter, 1.7% for active oncohematological disease, and 3% for TPN. Finally, the probability of CABSIs in patients with all five risk factors was 42.1%. The probabilities for developing CABSIs with all the combinations of factors are shown in Appendix A.

### 3.4. Validation of the Prognostic Equation

Among the 1549 patients included in the validation cohort, the frequency of CABSIs was 1.9% (n = 29). Multivariate analysis of this cohort identified the following as predictors of the development of CABSIs: having had bacteremia within a period of <3 months before catheter insertion (OR 2.53, 95% CI 1.10–5.85, *p* = 0.029), active oncohematological disease (OR 3.78, 95% CI 1.05–13.58, *p* = 0.041) and TPN (OR 5.53, 95% CI 2.49–12.30, *p* < 0.001). Tracheostomy was almost significant (OR 2.39, 95% CI 0.94–6.12, *p* = 0.06) and the only factor that was not related to CABSIs was having a synchronic central catheter (OR 1.78, 95% CI 0.62–5.09, *p* = 0.283).

This validation model obtained a good goodness-of-fit (Hosmer–Lemeshow test, *p* = 0.669) and an elevated predictive power for CABSIs (AUC = 0.77, 95% CI 0.68–0.87) (Table 5).

### 3.5. Decision Curve Analysis (DCA)

**Figure 2:**
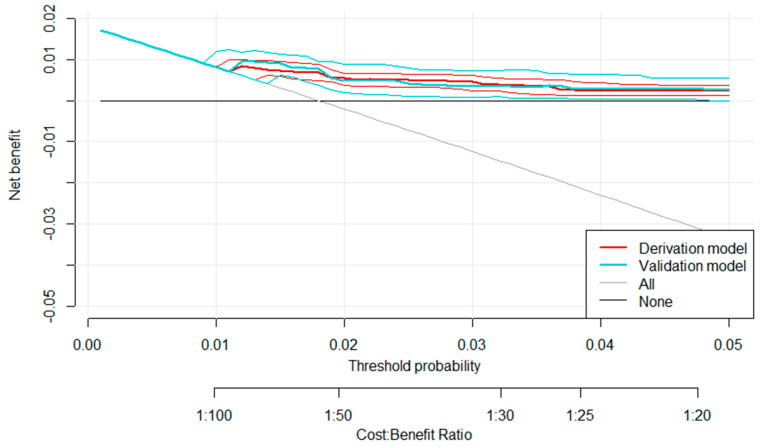
Decision curve analysis (DCA) comparing the derivation and validation models.

The decision curve analysis (DCA) demonstrated that both the derivation and validation models provided a higher net benefit than the “treat-all” and “treat-none” strategies across a range of threshold probabilities between 0.5% and 5%. The curves for the derivation and validation cohorts overlapped closely, indicating consistent model performance and potential clinical usefulness for guiding catheter-related infection prevention decisions (Figure 2).

### 3.6. Microbiology

The most prevalent microorganisms identified were Gram-positive bacteria, with *Staphylococcus epidermidis* constituting 44.2%, followed by *S. aureus* at 10.9%. Gram-negative bacteria were the second most numerous, with *Pseudomonas aeruginosa* and *Klebsiella* spp. constituting 8% and 6.5%, respectively. Lastly, the only *fungi* present were *Candida* spp. at 10.1% (Table 6).

## 4. Discussion

In the present study, a predictive model of CABSIs was developed in a large homogeneous sample of patients with midline catheters, CICC, and PICC. The results showed that 1.8% of the cases developed CABSIs, and the following independent risk factors were identified: TPN, active oncohematological disease, bacteremia within a period of less than 3 months before the insertion of a new catheter, the use of a synchronous central catheter, and the presence of tracheostomy. In addition, the coexistence of the five factors increased the probability of developing CABSIs to 42.1%. This predictive model is a tool that can be used to calculate the probability of developing CABSIs based on different combinations of these five risk factors (Appendix A). Given the low incidence of CLABSIs (1.8%), the model achieved a very high negative predictive value (98.8–99.2%) over thresholds between 1% and 5%, indicating excellent ability to rule out patients at low risk of infection. Although the positive predictive value was modest (3–9%), this is expected in low-prevalence outcomes. The model therefore provides clinical utility primarily as a rule-out tool, helping to identify patients unlikely to develop CLABSIs, which is particularly relevant in settings with limited resources.

Regarding the independent risk factors in our cohort, TPN had the greatest impact on the development of CABSIs. Previous studies and a recent meta-analysis also identified TPN as an independent factor for CABSIs [12]. The increased risk of CABSIs associated with TPN is related to higher dextrose serum concentrations, which promote microorganism proliferation [20,21,22]. Catheter dwell time and TPN, as well as the quality of catheter manipulation by health professionals, also impact the development of infection [15,23].

Similar to the results of previous studies, our predictive model identified active oncohematological disease as an independent risk factor for CABSIs [12]. Patients with this condition undergo chemotherapy, which can weaken the immune system and has been recognized as a risk factor in other studies [21]. Although a systematic review and meta-analysis did not explicitly classify it as a risk factor, oncohematological disease is associated with immune system compromise and chemotherapy, both of which have been reported as risk factors [24].

The presence of bacteremia within 3 months prior to the insertion of a new venous PICC, CICC, or midline catheter has been associated with the risk of developing CABSIs. This is related to the presence of residual microorganisms that facilitate catheter colonization by hematogenous spread [4]. Catheter-associated infections may recur in up to 41.5% of patients who have had CABSIs, particularly in those with fungal infections and ICU stays [22]. In our predictive model, the presence of a synchronous central catheter was also associated with the risk of CABSIs, especially in patients on hemodialysis and those requiring intensive care. Patients receiving hemodialysis have various risk factors, such as vascular access for renal replacement therapy, immune compromise, antibiotic resistance, comorbidities such as diabetes, and nasal colonization by *S. aureus* [25,26]. Critically ill patients often require more than one central venous catheter due to extensive pharmacotherapy and monitoring of hemodynamic parameters. Having two or more catheter insertion sites and increasing the number of lumens heightens the risk of CABSIs [24,27]. While tracheostomy was not statistically significant in the validation cohort, it remained clinically relevant and warrants further investigation. Previous studies suggest a potential link between CABSIs and tracheostomy. Biologically, tracheostomy may increase CABSI risk by promoting bacterial colonization in the upper airway, increasing secretion burden and manipulation, and indicating a higher severity of illness that necessitates prolonged hospitalization and invasive care. However, the limited sample size and observational designs of existing studies have prevented tracheostomy from being established as a definitive risk factor [23,28].

A predictive tool was developed from these risk factors. This calculator effectively identifies patients at higher risk for CABSIs, facilitating the implementation of preventive strategies to reduce potential infectious complications. The tool also allows personalization of surveillance and follow-up protocols to meet individual patients’ needs, thereby optimizing clinical resources and improving healthcare quality. DCA further demonstrated that both the derivation and validation models provided a greater net clinical benefit than the “treat-all” and “treat-none” strategies over a range of threshold probabilities between 0.5% and 5%. This supports the clinical applicability and consistency of the model, confirming its potential to guide real-world decision-making in catheter-related infection prevention. This personalization not only enhances patient prognosis but also leads to more efficient and proactive clinical risk management. In addition, the development of CABSIs significantly impacts hospital costs, with reported expenses of up to €18,000 per episode [29].

Beyond its predictive value, this tool can be integrated into electronic records to generate real-time alerts, assisting clinical teams in determining the appropriate type of line and the optimal timing of insertion based on each patient’s individual risk. This approach not only measures risk but also directly translates into specific actions within the clinical workflow and the preventive strategy of the infection control team. Importantly, the development of CABSIs is multifactorial, with some factors being preventable. Therefore, the use of various strategies, sometimes simultaneously, is essential to reduce the risk of CABSIs. A critical aspect in this context is promoting a centralized model for vascular access management through VATs. Several prospective studies have confirmed the efficacy of VATs in reducing catheter-related complications and their economic benefits [30,31,32,33,34]. This low prevalence may be partly related to the involvement of the VAT in catheter insertion and management; however, given the absence of a comparator group, this finding should be interpreted as a potential contributing factor rather than a causal explanation.

The microbiological profile observed in our cohort was characterized by a predominance of Gram-positive microorganisms, particularly *Staphylococcus epidermidis*, which is consistent with the pathogenesis of catheter-related bloodstream infections and the role of skin colonization in catheter contamination [23]. Gram-negative bacilli and *Candida* spp. were also identified, supporting the clinical relevance of host-related factors, severity of illness, and exposure to invasive procedures or parenteral nutrition in this population [12,20,21,22].

One recommended strategy for reducing infections related to the management of venous catheters is to implement or strengthen control policies that encourage early catheter withdrawal or avoid inserting non-essential venous catheters [15]. Collaboration with antibiotic optimization programs would facilitate early withdrawal of endovenous devices in patients eligible for oral antibiotic treatment, based on bioavailability, oral tolerance, and good clinical progress [27]. Increasing adherence among professionals to established protocols would improve venous catheter management and care. In this context, a phenomenological study found that resource limitations and the lack of intuitive procedures contribute to low adherence [35]. Therefore, qualitative studies are needed for an in-depth exploration of the causes related to low adherence to institutional protocols among professionals.

Other more specific strategies that can be applied to prevent CABSIs of endoluminal origin include the use of antiseptic solutions such as taurolidine [15,36], venous catheters impregnated with antimicrobials [37,38], and passive disinfection with disinfectant caps to enhance adherence to bioconnector disinfection [15,39]. Chlorhexidine-impregnated dressings can also be used to protect the insertion site and prevent CABSIs of extraluminal origin [15].

As a main strategy, it is recommended to develop integrated bundles that implement these strategies and evaluate their efficacy and efficiency in randomized clinical trials. These bundles can be tailored to the risks associated with different devices, interventions, and management models, supported by strong scientific evidence, to prevent CABSIs. In addition, this approach would facilitate the implementation of educational resources to improve adherence to evidence-based practices among professionals.

The development of future pathogen identification lines and consideration of associated microbiota would optimize the predictive model, enhancing its accuracy in identifying specific risks and personalizing interventions. This would also offer valuable insights for nursing research, guiding studies on infection prevention and patient care strategies. Such research would improve the adaptation of the model to distinct clinical settings, patient populations, and epidemiological scenarios, promoting more effective prevention strategies tailored to the particular needs of each context.

## 5. Limitations

The overall incidence of CLABSIs in our cohort was 1.8%, consistent with previously reported rates. Although the total sample was large, the low number of events may have limited the statistical power to detect modest associations and could affect model calibration. To reduce potential overfitting, the number of predictors included in the final model was restricted, and internal validation using an independent cohort showed acceptable calibration and discrimination. In addition, the exclusion of patients admitted in 2020 may limit the temporal generalizability of the findings and represents a potential source of selection bias. This predictive model was developed at a single center, and its performance may vary in hospitals without a VAT or other specialized infrastructure; therefore, external validation in diverse populations will be necessary in future studies. Nevertheless, the model was internally validated with a randomly selected cohort. The loss of some data, due to reliance on other professionals for recording variables in electronic medical records, may also have affected the analysis; these missing values were processed using a statistically robust imputation method, described in detail in the Methods Section. Although multiple imputation was performed, the main analyses were based on a single imputed dataset rather than on pooled estimates using Rubin’s rules, which may have underestimated variability. However, results were consistent across imputations, supporting the robustness of the findings. Furthermore, an important confounding variable, repeated among the different objectives of this work, is the quality of manipulation and handling of venous devices. However, our institution has a periodic training program, designated care leaders in each hospitalization unit, and a VAT that routinely reinforces proper management of venous devices. In addition, this study is confined to the setting of venous devices; therefore, its findings should be interpreted with caution and cannot be directly generalized to all cases of CLABSI or to other clinical contexts unrelated to venous access.

Despite the limitations mentioned above, this study identified factors related to CABSIs by robust statistical methods. The validation of the model and the development of a user-friendly prognostic calculator contribute positively to clinical outcomes. It is important to note that the causes of CABSIs are multifactorial, and the prognostic calculator developed in this study can identify patients at risk for CABSIs before venous catheter insertion.

## 6. Conclusions

This study identified five factors independently associated with the development of CABSIs and enabled the development of a prognostic model with potential clinical utility for estimating risk before catheter insertion. The presence of total parenteral nutrition, active hematologic malignancy, bacteremia within the three months prior to catheter insertion, the concurrent use of another central venous catheter, and tracheostomy was associated with a relevant increase in the risk of CABSIs. Based on these findings, the developed tool may help stratify risk on an individual basis and support preventive decision-making, including the optimization of device selection, the timing of insertion, the intensity of care, and the implementation of preventive measures tailored to each patient’s clinical profile. In addition, decision curve analysis supported the clinical utility of the model by showing a net benefit across a clinically relevant range of threshold probabilities. This approach may also reinforce the role of vascular access teams in the prevention of catheter-related infections and in improving patient safety. External studies will be needed to confirm the validity and applicability of the model in other clinical settings.

## 7. Summary Box

### 7.1. What Problem Did the Study Address?

- The existing literature has identified evidence of various precursor risk factors of CABSIs, but the findings are heterogeneous and inconclusive. Moreover, these studies did not include midline catheters, which have gained increasing clinical relevance in recent years.
- Investigation of CABSI risk factors supports the development of evidence-based insertion protocols and care strategies, equipping healthcare professionals with targeted guidelines to prevent CABSIs and reduce associated morbidity, mortality, length of hospital stay, and healthcare costs.
- By identifying independent risk factors and developing a predictive model, this study allows the design of personalized care plans for vulnerable patients while improving the efficiency of hospital resource use.
- The inclusion of decision curve analysis (DCA) demonstrated that both the derivation and validation models provided a higher net clinical benefit than the “treat-all” and “treat-none” strategies at threshold probabilities ranging from 0.5% to 5%. The overlapping curves between the derivation and validation cohorts confirmed consistent model performance and its potential clinical applicability in guiding catheter-related infection prevention strategies.

### 7.2. What Were the Main Findings?

Five variables were independently associated with CABSIs: tracheostomy (1.5% probability), bacteremia within <3 months before catheter insertion (1.5%), the presence of a synchronous central-line catheter (1.5%), active oncohematological disease (1.7%), and receipt of TPN (3%). The probability of CABSIs in the absence of these factors was 0.7%, while the presence of all five factors increased the risk to 41.2%. The predictive model demonstrated strong performance (AUC 0.73 in the derivation cohort; 0.77 in the validation cohort) and underwent internal validation using a split-sample approach. DCA confirmed its robustness and clinical usefulness, supporting its potential to improve decision-making in catheter-related infection prevention.

### 7.3. Where and on Whom Will the Research Have an Impact?

The study is relevant to all patients with central venous or midline catheters, as venous access remains the most frequently performed invasive technique in hospitals. By incorporating DCA into model validation, the model provides clinicians with a practical, evidence-based framework to weigh the benefits and risks of preventive interventions. Its application has the potential to optimize patient outcomes and enhance healthcare resource utilization.

## Figures and Tables

**Table 1:** Categorical variables by cohort. Descriptive statistics. Total study population.

VARIABLE	TOTAL (N = 7585)	DERIVATION (N = 6036)	VALIDATION (N = 1549) {XE “VARIABLE”}	*p*-VALUE
DEMOGRAPHIC VARIABLES				
Sex n (%)				
Male	4478 (59)	3559 (59)	919 (59.3)	0.79
Female	3107 (41)	2477 (41)	630 (40.7)	
Age	71 (15.99)	71 (16)	71 (15.94)	0.38
HOSPITALIZATION UNIT n (%)				
Critical care	3092 (40.8)	2474 (41)	618 (39.9)	
Conventional hospitalization	4483 (59.1)	3557 (58.9)	926 (59.8)	0.15
Home hospitalization	9 (0.1)	5 (0.1)	4 (0.3)	
PATHOLOGICAL HISTORY				
Bacteremia < 3 months n (%)	1145 (15.1)	918 (15.2)	227 (14.7)	0.58
Active oncohematological disease n (%)	369 (4.9)	290 (4.8)	79 (5.1)	0.63
Charlson index	5.55 (3.32)	5.57 (3.33)	5.48 (3.26)	0.51
HEMODIALYSIS n (%)	284 (3.7)	221 (3.7)	63 (4.1)	0.45
INVASIVE MECHANICAL VENTILATION n (%)	925 (12.2)	732 (12.1)	193 (12.5)	0.72
SEPTIC SHOCK n (%)	404 (5.3)	312 (5.2)	92 (5.9)	0.22
TEMPORALITY				
Days of hospitalization until catheter insertion	11.18 (28.78)	10.91 (28.89)	12.22 (28.34)	0.053
Days with catheter	6.28 (9.81)	6.3 (9.24)	6.2 (11.82)	0.32
PHARMACOTHERAPY n (%)				
Total parenteral nutrition	1563 (20.3)	1207 (20)	329 (21.2)	0.27
TYPE OF CATHETER n (%)				
CICC	3727 (49.1)	2961 (49.1)	766 (49.5)	
Midline	1935 (25.5)	1547 (25.6)	388 (25)	0.89
PICC	1923 (25.4)	1528 (25.3)	395 (25.5)	
CATHETER CHARACTERISTICS				
Number of lumens	2.34 (1.12)	2.35 (1.13)	2.33 (1.1)	0.86
CATHETER HISTORY n (%)				
Synchronic central catheter	431 (5.7)	336 (5.6)	95 (6.1)	0.39
History of central venous catheter	3085 (40.7)	2434 (40.3)	651 (42)	0.22
Number of peripheral catheters prior to catheter placement.	1.75 (2.58)	1.73 (2.56)	1.83 (2.68)	0.30
CABSI	136 (1.8)	107 (1.8)	29 (1.9)	0.79

NOTE: The data are expressed as mean and standard deviation unless otherwise specified; CICC, centrally inserted central catheter; PICC, peripherally inserted central catheter; CABSI, catheter-associated bloodstream infection; NLCR, neutrophil-to-lymphocyte ratio; CRP, C-reactive protein. The complete version of this table is provided in Supplementary Table S1.

**Table 2:** Patient and catheter characteristics according to the presence of CABSIs. Derivation cohort.

VARIABLE	TOTAL (N = 6036)	NO CABSI (N = 5929)	CABSI (N = 107)	*p*-VALUE
DEMOGRAPHIC VARIABLES				
Sex n (%)				
Male	3559 (59%)	3498 (59%)	61 (57%)	0.67
Female	2477 (41%)	2431 (41%)	46 (43%)	
Age	68.42 (16)	68.43 (16.01)	68.27 (15.56)	0.89
TYPE OF PATIENT n (%)				
Critical	2474 (41%)	2431 (41%)	43 (40.2%)	
Conventional hospitalization	3557 (58.9%)	3493 (58.9%)	64 (59.8%)	0.94
Home hospitalization	5 (0.1%)	5 (0.1%)	0 (0%)	
PATHOLOGICAL HISTORY				
Bacteremia < 3 months n (%)	918 (15.2%)	882 (14.9)	36 (33.6)	<0.001
Active oncohematological disease n (%)	290 (4.8%)	279 (4.7%)	11 (10.3%)	0.008
Charlson index	5.57 (3.33%)	5.57 (3.34%)	5.1 (3.28%)	0.153
HEMODIALYSIS n (%)	221 (3.7%)	210 (3.5%)	11 (10.3%)	0.002
INVASIVE MECHANICAL VENTILATION n (%)	732 (12.1%)	706 (11.9%)	26 (24.3%)	<0.001
SEPTIC SHOCK n (%)	312 (5.2%)	304 (5.1%)	8 (7.5%)	0.277
TRACHEOSTOMY n (%)	632 (10.5%)	611 (10.3%)	21 (19.6%)	0.002
TEMPORALITY				
Days of hospitalization until catheter insertion	10.91 (28.89)	10.43 (26.46)	37.49 (87.84)	<0.001
Days with catheter	6.3 (9.24)	6.13 (8.84)	13.71 (18.84)	0.004
PHARMACOTHERAPY n (%)				
Total parenteral nutrition	1207 (20%)	1148 (19.4%)	59 (55.1%)	<0.001
TYPE OF CATHETER n (%)				
CICC	2961 (49.1%)	2912 (49.1%)	49 (45.8%)	<0.001
Midline	1547 (25.6%)	1533 (25.9%)	14 (13.1%)	
PICC	1528 (25.3%)	1484 (25%)	44 (41.1%)	
CATHETER CHARACTERISTICS				
Number of lumens	2.35 (1.13)	2.34 (1.12)	2.69 (1.15)	0.002
Length of catheter	27.22 (15.77)	27.17 (15.8)	30.49 (13.66)	0.005
Catheter-to-vein ratio	32.92 (5.42)	32.91 (5.42)	33.82 (5.72)	0.523
CATHETER HISTORY n (%)				
Synchronic central catheter	336 (5.6%)	316 (5.3%)	20 (18.7%)	<0.001
Central-line catheter	2434 (40.3%)	2370 (40%)	64 (59.8%)	<0.001
Number of peripheral catheters prior to catheter placement.	1.73 (2.56)	1.7 (2.52)	3.31 (3.71)	<0.001

NOTE: The data are expressed as mean and standard deviation unless otherwise specified.; CICC, centrally inserted central catheter; PICC, peripherally inserted central catheter; CABSI, catheter-associated bloodstream infection; NLCR, neutrophil-to-lymphocyte ratio; The complete version of this table is provided in Supplementary Table S2.

**Table 3:** Univariate logistic regression analysis of variables associated with catheter-associated bloodstream infection (CABSI) ^a^.

Variable ^b^	OR	95% CI	*p*-Value
Albumin day of insertion (+1)	0.46	0.35–0.61	<0.001
Number of days of ICU + RES stay (+1)	1.02	1.01–1.02	<0.001
Number of peripheral vias ≥ 2	2.13	1.45–3.13	<0.001
Number of lumens ≥ 2	2.13	1.27–3.59	0.004
Tracheostomy	2.13	1.31–3.45	0.002
Central catheter	2.24	1.51–3.30	<0.001
Active oncohematological disease	2.32	1.23–4.38	0.009
Invasive mechanical ventilation ^c,d^	2.37	1.52–3.72	<0.001
Peripheral parenteral nutrition	2.59	1.18–5.67	0.017
Hemodialysis catheter ^c,d^	2.83	1.13–7.09	0.027
Bacteremia < 3 months before insertion	2.90	1.93–4.36	<0.001
Hemodialysis	3.12	1.65–5.91	<0.001
Synchronic central catheter	4.08	2.48–6.73	<0.001
Total parenteral nutrition	5.12	3.48–7.53	<0.001

NOTE: ICU, intensive care unit; RES, resuscitation unit; OR: odds ratio; CI, confidence interval. ^a^ Hosmer–Lemeshow goodness-of-fit test; *p* = 0.735. ^b^ Area under the ROC curve AUC = 0.73 (95% CI: 0.67 to 0.78). ^c^ AUC = 0.71 (95% CI: 0.66 to 0.77. ^d^ Excluded from the multivariate model due to a high correlation with another variable (r > |±0.6|).

**Table 4:** Logistic regression analysis of variables associated with catheter-associated bloodstream infection (CABSI) ^a^.

Variable ^b^	OR	95% CI	*p*-Value	β Coefficient ^b^	Non-Weighted Score ^c^	Weighted Score ^d^
Albumin day of insertion (+1)	-	-		-	-	-
Number of days of ICU + RES stay (+1)	-	-	-	-	-	-
Number of peripheral catheters prior to catheter placement.	-	-	-	-	-	-
Number of lumens ≥ 2	-	-	-	-	-	-
Tracheostomy	1.85	1.12–3.06	0.017	0.61	1	6
Central catheter	-	-	-		-	-
Active oncohematological disease	2.56	1.33–4.92	0.005	0.94	1	9
Invasive mechanical ventilation ^c,e^	-	-	-	-	-	-
Peripheral parenteral nutrition	-	-	-	-	-	-
Hemodialysis catheter ^c,e^	-	-	-	-	-	-
Bacteremia < 3 months before insertion	2.20	1.43–3.37	<0.001	0.79	1	8
Hemodialysis	-	-	-	-	-	-
Synchronic central catheter	2.25	1.32–3.85	0.003	0.81	1	8
Total parenteral nutrition	4.47	3.00–6.64	<0.001	1.50	1	15

NOTE: ICU, intensive care unit; RES, resuscitation unit; OR: odds ratio; CI, confidence interval. ^a^ Hosmer–Lemeshow goodness-of-fit test; *p* = 0.735. ^b^ Area under the ROC curve AUC = 0.73 (95% CI: 0.67 to 0.78). ^c^ AUC = 0.71 (95% CI: 0.66 to 0.77). ^d^ Area under the ROC curve, AUC = 0.73 (95% CI: 0.67 to 0.78). ^e^ Excluded from the multivariate model due to a high correlation with another variable (r > |±0.6|).

**Table 5:** Validation of the predictive model for CABSIs. Validation cohort (N = 1549).

	Multivariate ^a^		
Variable ^b^	OR	95% CI	*p*-Value
Bacteremia < 3 months	2.53	1.10–5.85	0.029
Synchronic central catheter	1.78	0.62–5.09	0.283
Tracheostomy	2.39	0.94–6.12	0.068
Active hematological neoplastic disease	3.78	1.05–13.58	0.041
Total parenteral nutrition	5.53	2.49–12.30	<0.001

NOTE: OR indicates odds ratio; CI, confidence interval. Data are shown as estimated ORs (95% CIs) of the explanatory variables in the CLABSI group. The OR represents the odds that the presence of CLABSIs will occur given exposure of the explanatory variable, compared to the odds of the outcome occurring in the absence of that exposure; for continuous predictors, the OR represents the increase in odds of the outcome of interest with every one unit increase in the input variable. The *p*-values are based on the null hypothesis that all ORs relating to an explanatory variable equal unity (no effect). ^a^ Hosmer–Lemeshow goodness-of-fit test, *p* = 0.669. Area under the ROC curve, AUC = 0.77 (95% CI 0.68 to 0.87). ^b^ Multiple imputation method was used for missing data.

**Table 6:** Microbiological results of the total number of catheter-associated bloodstream infections (CABSIs).

	N = 138	%
Gram-Positive Microorganisms		
*Staphylococcus aureus*	15	10.9
*Staphylococcus epidermidis*	61	44.2
Other *Staphylococcus coag* (−)	8	5.8
*Enterococcus* spp.	8	5.8
*Corynebacterium* spp.	1	0.7
Subtotal	93	
*Gram-negative*		
*Escherichia coli*	2	1.4
*Enterobacter cloacae*	4	2.9
*Klebsiella* spp.	9	6.5
*Pseudomonas aeruginosa*	11	8.0
*Proteus mirabillis*	2	1.4
*Serratia marcescens*	2	1.4
*Stenotrophomonas maltophila*	1	0.7
Subtotal	31	
*Fungi*		
*Candida* spp.	14	10.1
*Total*	138	100.0

## Data Availability

The data presented in this study are not publicly available due to privacy and ethical restrictions, as they contain sensitive information from human participants. Access to the data may be considered upon reasonable request to the corresponding author and subject to approval by the relevant Ethics Committee.

## Supplementary Materials

The following supporting information can be downloaded at: https://www.mdpi.com/article/10.3390/jcm15093243/s1, Table S1. Categorical variables by cohort. Descriptive statistics. Total study population; Table S2. Patient and catheter characteristics according to the presence of CABSI. Derivation cohort.

## Appendix A. Table of Probability of Developing CABSI Based on the Presence of the Different Risk Factors

**Bacteraemia < 3 Months**	**Synchronic Central Catheter**	**Total Parenteral Nutrition**	**Active Haematological Neoplastic Disease**	**Tracheostomy**	**PROBABILITY OF CABSI %**
NO	NO	NO	NO	NO	**0.69%**
NO	NO	NO	NO	**YES**	**1.27%**
**YES**	NO	NO	NO	NO	**1.50%**
NO	**YES**	NO	NO	NO	**1.54%**
NO	NO	NO	**YES**	NO	**1.74%**
NO	NO	**YES**	NO	NO	**3.00%**
**YES**	NO	NO	NO	**YES**	**2.74%**
NO	**YES**	NO	NO	**YES**	**2.81%**
**YES**	**YES**	NO	NO	NO	**2.81%**
NO	NO	NO	**YES**	**YES**	**3.18%**
**YES**	NO	NO	**YES**	NO	**3.75%**
NO	**YES**	NO	**YES**	NO	**3.85%**
NO	NO	**YES**	NO	**YES**	**5.42%**
**YES**	NO	**YES**	NO	NO	**6.37%**
NO	**YES**	**YES**	NO	NO	**6.53%**
NO	NO	**YES**	**YES**	NO	**7.34%**
**YES**	**YES**	NO	NO	**YES**	**5.97%**
**YES**	NO	NO	**YES**	**YES**	**6.73%**
NO	**YES**	NO	**YES**	**YES**	**6.89%**
**YES**	**YES**	NO	**YES**	NO	**8.08%**
**YES**	NO	**YES**	NO	**YES**	**11.17%**
NO	**YES**	**YES**	NO	**YES**	**11.44%**
NO	NO	**YES**	**YES**	**YES**	**12.79%**
**YES**	**YES**	**YES**	NO	NO	**13.30%**
**YES**	NO	**YES**	**YES**	NO	**14.83%**
NO	**YES**	**YES**	**YES**	NO	**15.16%**
**YES**	**YES**	**YES**	**YES**	**YES**	**13.99%**
**YES**	**YES**	**YES**	NO	**YES**	**22.10%**
**YES**	NO	**YES**	**YES**	**YES**	**24.36%**
NO	**YES**	**YES**	**YES**	**YES**	**24.84%**
**YES**	**YES**	**YES**	**YES**	NO	**28.19%**
**YES**	**YES**	**YES**	**YES**	YES	**42.07%**
