# Supplementary material for: Risk Factor Prediction Model for Catheter-Associated Bloodstream Infections (CABSIs) in Midline and Central Venous Catheters: A Cohort Follow-Up Study
Source: J Clin Med. 2026 Apr 24;15(9):3243. doi: 10.3390/jcm15093243 (PMC13164023; doi:10.3390/jcm15093243)
Supplement: Supplementary file 1 [file jcm-15-03243-s001.zip › jcm-4193680-supplementary.pdf]

Table S1. Categorical variables by cohort. Descriptive statistics. Total study population.

VARIABLE	TOTAL (N=7,585)	DERIVATION (N=6,036)	VALIDATION (N=1,549)	P-VALUE
**DEMOGRAPHIC VARIABLES**				
**Sex n (%)**				
Male	4,478 (59)	3,559 (59)	919 (59.3)	0.79
Female	3107(41)	2,477 (41)	630 (40.7)	
Age	71 (15.99)	71 (16)	71 (15.94)	0.38
**HOSPITALIZATION UNIT n (%)**				
Critical care	3,092 (40.8)	2,474(41)	618 (39.9)	
Conventional hospitalization	4,483 (59.1)	3,557 (58.9)	926 (59.8)	0.15
Home hospitalization	9 (0.1)	5 (0.1)	4 (0.3)	
**PATHOLOGICAL HISTORY**				
Bacteraemia <3 months n (%)	1,145 (15.1)	918 (15.2)	227(14.7)	0.58
Coronary disease n (%)	758 (10)	623 (10.3)	135 (8.7)	0.06
Congestive heart disease n (%)	1,027 (13.5)	826 (13.7)	201 (13)	0.47
Peripheral vascular disease n (%)	1,383 (18.2)	1,080 (17.9)	303 (19.6)	0.12
Cerebrovascular disease n (%)	948 (12.5)	791 (13.1)	157 (10.1)	**<0.001**
Dementia n (%)	395 (5.2)	318 (5.3)	77 (5)	0.64
COPD n (%)	1,446 (19.1)	1,148 (19)	298 (19.3)	0.84
Connective tissue disease (%)	287 (3.8)	225 (3.7)	62 (4)	0.61
Peptic ulcer n (%)	282 (3.7)	231 (3.8)	51(3.3)	0.32
Liver disease n (%)	1,083 (14.3)	867 (14.4)	216 (14)	0.67
Diabetes mellitus n (%)	2,252 (29.7)	1,779 (29.5)	473 (30.6)	0.41
Hemiplegia n (%)	125 (1.6)	89 (1.5)	36 (2.3)	**0.01**
Kidney disease n (%)	1,417 (18.7)	1,137 (18.8)	280 (18.1)	0.49
Neoplasia n (%)	2,393 (31.6)	1,893 (31.4)	500 (32.3)	0.48
Metastatic disease n (%)	651 (8.6)	524 (8.7)	127 (8.2)	0.54
HIV n (%)	194 (2.6)	160 (2.7)	34 (2.2)	0.31
Active oncohaematological disease n (%)	369 (4.9)	290 (4.8)	79 (5.1)	0.63
Charlson index	5.55 (3.32)	5.57 (3.33)	5.48 (3.26)	0.51
BMI n (%)	27.45 (6.81)	27.48 (6.81)	27.33 (6.8)	0.52
**HAEMODIALYSIS** n (%)	284 (3.7)	221(3.7)	63 (41)	63 (41)
**EMERGENCY SURGICAL INTERVENTION** n (%)	98 (1.3)	77 (1.3)	21 (1.4)	0.80
**ICU/RES STAY** n (%)	4,353 (57.4)	3,455 (57.2)	898 (58)	0.60
**INVASIVE MECHANICAL VENTILATION** n (%)	925 (12.2)	732 (12.1)	193 (12.5)	0.72
**SEPTIC SHOCK** n (%)	404 (5.3)	312 (5.2)	92 (5.9)	0.22
**TEMPORALITY**				
Days of hospitalization until catheter insertion	11.18 (28.78)	10.91 (28.89)	12.22 (28.34)	**0.053**
Days with catheter	6.28 (9.81)	6.3 (9.24)	6.2 (11.82)	0.32
**SEASON OF THE YEAR n (%)**				
Summer	1,793 (23.6)	1,433 (23.7)	360 (23.2)	
Winter	1,943 (25.6)	1,570 (26)	373 (24.1)	
Fall	2,081 (27.4)	1,634 (27.1)	447 (28.9)	0.31
Spring	1,768 (23.3)	1,399 (23.2)	369 (23.8)	
**PHARMACOTHERAPY n (%)**				
Vasoactive drugs	440 (5.8)	350 (5.8)	90 (5.8)	0.98
Peripheral parenteral nutrition	203 (2.7)	163 (2.7)	40 (2.6)	0.79

Total parenteral nutrition	1,563 (20.3)	1,207 (20)	329 (21.2)	0.27
Serum therapy	1,457 (19.2)	1,182 (19.6)	275 (17.8)	0.10
Oral supplements	784 (10.3)	628 (10.4)	156 (10.1)	0.70
Immunosuppressive treatment	925 (12.2)	748 (12.4)	177 (11.4)	0.30
Number of manipulations/24 hours	13 (13.89)	13 (13.89)	13 (13.93)	0.32
**TYPE OF CATHETER n (%)**				
CICC	3,727 (49.1)	2,961 (49.1)	766 (49.5)	0.89
Midline	1,935 (25.5)	1,547 (25.6)	388 (25)	
PICC	1,923 (25.4)	1,528 (25.3)	395 (25.5)	
**CATHETER CHARACTERISTICS**				
Number of lumens	2.34 (1.12)	2.35 (1.13)	2.33 (1.1)	0.86
Catheter length	27.22 (15.77)	27.17 (15.8)	30.49 (13.66)	**0.005**
Catheter-to-vein ratio	32.94 (5.44)	32.92 (5.42)	33.04 (5.56)	0.82
**INSERTION CHARACTERISTICS**				
**Vein n (%)**				
Axillary	74 (1)	59 (1)	15 (1)	0.35
Basilic	3,573 (47.1)	2,863 (47.5)	710 (45.8)	
Brachial	690 (9.1)	546 (9.1)	144 (9.3)	
Cephalic	501 (6.6)	407 (6.7)	94 (6.1)	
Femoral	205 (2.7)	161 (2.7)	44 (2.8)	
Jugular	2,079 (27.4)	1,650 (27.3)	429 (27.7)	
Subclavian	460 (6.1)	347 (5.8)	113 (7.3)	
**CATHETER HISTORY n (%)**				
Synchronic central catheter	431 (5.7)	336 (5.6)	95 (6.1)	0.39
History of central venous catheter	3,085 (40.7)	2,434 (40.3)	651 (42)	0.22
Number of peripheral vías prior to catheter placement	1.75 (2.58)	1.73 (2.56)	1.83 (2.68)	0.30
**CABSI**	136 (1.8)	107 (1.8)	29 (1.9)	0.79
**ANALYTICAL PARAMETERS**				
Albumin	3.08 (0.73)	3.08 (0.71)	3.11 (0.77)	0.62
Eosinophils	0.14 (0.23)	0.13 (0.22)	0.15 (0.25)	0.38
Leukocytes	12.23 (8.78)	12.11 (7.9)	12.72 (11.62)	0.65
NLCR	11.58 (15.33)	11.67 (15.52)	11.21 (14.52)	0.14
Neutrophils	11.78 (15.29)	11.84 (15.55)	11.53 (14.22)	0.43
CRP	10.69 (11.76)	10.6 (11.77)	11 (11.73)	0.28
Procalcitonin	12.93 (45.36)	13.28 (45.59)	11.58 (44.48)	**0.01**

** NOTE: The data are expressed as mean and standard deviation unless otherwise specified; COPD, chronic obstructive pulmonary disease; HIV, human immunodeficiency virus; ICU, intensive care unit; RES, resuscitation; BMI, body mass index; CICC, centrally inserted central catheter; PICC, peripherally inserted central catheter; CABSI, catheter-associated bloodstream infection.; NLCR, neutrophil-to-lymphocyte ratio; CRP, C-reactive protein.

Table S2. Patient and catheter characteristics according to the presence of CABSI. Derivation cohort.

VARIABLE	TOTAL (N=6036)	NO CABSI (N=5929)	CABSI (N=107)	P-VALUE
**DEMOGRAPHIC VARIABLES**				
Sex n (%)				
Male	3,559 (59%)	3,498 (59%)	61 (57%)	0.67
Female	2,477 (41%)	2,431 (41%)	46 (43%)	
Age	68.42 (16)	68.43 (16.01)	68.27 (15.56)	0.89
**TYPE OF PATIENT n (%)**				
Critical	2,474 (41%)	2,431 (41%)	43 (40.2%)	0.94
Conventional hospitalization	3,557 (58.9%)	3,493 (58.9%)	64 (59.8%)	
Home hospitalization	5 (0.1%)	5 (0.1%)	0 (0%)	
**PATHOLOGICAL HISTORY**				
Bacteraemia <3 months n (%)	918(15.2%)	882 (14.9)	36 (33.6)	**<0.001**
Coronary disease n (%)	623 (10.3%)	613 (10.3%)	10 (9.3%)	0.738
CHF n (%)	826 (13.7%)	807 (13.6%)	19 (17.8%)	0.217
Peripheral artery disease n (%)	1,080 (17.9%)	1,066 (18%)	14 (13.1%)	0.190
Cerebrovascular disease n (%)	791 (13.1%)	782 (13.2%)	9 (8.4%)	0.146
Dementia n (%)	318 (5.3%)	309 (5.2%)	9 (8.4%)	0.142
COPD n (%)	1,148 (19%)	1,132 (19.1%)	16 (15%)	0.279
Connective tissue disease n (%)	225 (3.7%)	222 (3.7%)	3 (2.8%)	0.799
Peptic ulcer n (%)	231 (3.8%)	228 (3.8%)	3 (2.8%)	0.799
Liver disease n (%)	867 (14.4%)	859 (14.5%)	8 (7.5%)	0.040
Diabetes mellitus n (%)	1,779 (29.5%)	1,743 (29.4%)	36 (33.6%)	0.341
Haemiplegia n (%)	89 (1.5%)	87 (1.5%)	2 (1.9%)	0.672
Kidney disease n (%)	1,137 (18.8%)	1,120 (18.9%)	17 (15.9%)	0.430
Neoplasia n (%)	1,893 (31.4%)	1,856 (31.3%)	37 (34.6%)	0.471
Metastatic disease n (%)	524 (8.7%)	517 (8.7%)	7 (6.5%)	0.427
HIV n (%)	160 (2.7%)	159 (2.7%)	1 (0.9%)	0.533
Active oncohaematological disease n (%)	290 (4.8%)	279 (4.7%)	11 (10.3%)	**0.008**
Charlson index	5.57 (3.33%)	5.57 (3.34%)	5.1 (3.28%)	0.153
Body mass index n (%)	27.48 (6.81)	27.47 (6.81)	27.86 (6.87)	0.425
**HAEMODIALYSIS** n (%)	221 (3.7%)	210 (3.5%)	11 (10.3%)	**0.002**
**EMERGENCY SURGICAL INTERVENTION** n (%)	77 (1.3%)	76 (1.3%)	1 (0.9%)	0.999
**ICU/RES STAY** n (%)	3,455 (57.2%)	3,393 (57.2%)	62 (57.9%)	0.882
**INVASIVE MECHANICAL VENTILATION** n (%)	732 (12.1%)	706 (11.9%)	26 (24.3%)	<**0.001**
**SEPTIC SHOCK** n (%)	312 (5.2%)	304 (5.1%)	8 (7.5%)	0.277
**TRACHEOSTOMY** n (%)	632 (10.5%)	611 (10.3%)	21 (19.6%)	**0.002**
**TEMPORALITY**				
Days of hospitalization until catheter insertion	10.91 (28.89)	10.43 (26.46)	37.49 (87.84)	**<0.001**
Days with catheter	6.3 (9.24)	6.13 (8.84)	13.71 (18.84)	**0.004**
**SEASON OF THE YEAR n (%)**				
Summer	1,433 (23.7%)	1,406 (23.7%)	27 (25.2%)	**0.024**
Winter	1,570 (26%)	1,542 (26%)	28 (26.2%)	
Fall	1,634 (27.1%)	1,617 (27.3%)	17 (15.9%)	
Spring	1,399 (23.2%)	1,364 (23%)	35 (32.7%)	
**PHARMACOTHERAPY n (%)**				

Vasoactive drugs	350 (5.8%)	342 (5.8%)	8 (7.5%)	0.454
Peripheral parenteral nutrition	163 (2.7%)	156 (2.6%)	7 (6.5%)	**0.025**
Total parenteral nutrition	1,207 (20%)	1,148 (19.4%)	59 (55.1%)	**<0.001**
Serum therapy	1,182 (19.6%)	1,153 (19.4%)	29 (27.1%)	**0.048**
Oral supplements	628 (10.4%)	613 (10.3%	15 (14%)	0.217
Immunosuppressive treatment	748 (12.4%)	731 (12.3%)	17 (15.9%)	0.268
Number of manipulations/24 hours	13.93 (5.45)	13.93 (5.45)	13.97 (5.09)	0.90
**TYPE OF CATHETER n (%)**				
CICC	2,961 (49.1%)	2,912 (49.1%)	49 (45.8%)	**<0.001**
Midline	1,547 (25.6%)	1,533 (25.9%)	14 (13.1%)	
PICC	1,528 (25.3%)	1,484 (25%)	44 (41.1%)	
**CATHETER CHARACTERISTICS**				
Number of lumens	2.35(1.13)	2.34(1.12)	2.69(1.15)	**0.002**
Length of catheter	27.22 (15.77)	27.17 (15.8)	30.49 (13.66)	**0.005**
Catheter-to-vein ratio	32.92 (5.42)	32.91 (5.42)	33.82 (5.72)	0.523
**CHARACTERISTICS OF INSERTION VESSEL n (%)**				
Axillary	59 (1%)	58 (1%)	1 (0.9%)	
Basilic	2,863 (47.5%)	2,810 (47.4%)	53 (49.5%)	0.115
Brachial	546 (9.1%)	539 (9.1%)	7 (6.5%)	
Cephalic	407 (6.7%)	403 (6.8%)	4 (3.7%)	
Femoral	161 (2.7%)	156 (2.6%)	5 (4.7%)	
Jugular	1650 (27.3%)	1625 (27.4%)	25 (23.4%)	
Subclavian	347 (5.8%)	335 (5.7%)	12 (11.2%)	
**CATHETER HISTORY n (%)**				
Synchronic central catheter	336 (5.6%)	316 (5.3%)	20 (18.7%)	**<0.001**
Cenral-line catheter	2,434 (40.3%)	2,370 (40%)	64 (59.8%)	**<0.001**
Number of peripheral vias prior to catheter insertion	1.73 (2.56)	1.7 (2.52)	3.31 (3.71)	**<0.001**
**ANALYTICAL PARAMETERS**				
Albumin	3.08 (0.73)	3.08 (0.71)	2.7 (0.65)	**0.001**
Eosinophils	0.13 (0.22)	0.13 (0.22)	0.19 (0.26)	0.119
Leukocytes	11.96 (7.8)	11.95 (7.76)	12.5 (10.12)	0.447
NLCR	11.67 (15.52)	11.68 (15.61)	11.08 (9.33))	0.212
Neutrophils	11.84 (15.55)	11.87 (15.64)	10.29 (8.83)	0.838
CRP	10.6 (11.77)	10.62 (11.8)	9.83 (10.3)	0.961
Procalcitonin	13.28 (45.59)	13.34 (45.84)	10.39 (31.74)	0.112

** NOTE: The data are expressed as mean and standard deviation unless otherwise specified. CHF: congestive heart failure; COPD, chronic obstructive pulmonary disease; HIV, human immunodeficiency virus; ICU, intensive care unit; RES, resuscitation; CICC, centrally-inserted central catheter; PICC, peripherally inserted central catheter; CABSI, catheter-assocatied bloodstream infection; NLCR, neutrophil-to-lymphocyte ratio; CRP, C-reactive protein.
